# Admission Screening of Methicillin-Resistant *Staphylococcus aureus* with Rapid Molecular Detection in Intensive Care Unit: A Three-Year Single-Centre Experience in Hong Kong

**DOI:** 10.1155/2013/140294

**Published:** 2013-09-19

**Authors:** Eddie Chi Man Leung, May Kin Ping Lee, Raymond Wai Man Lai

**Affiliations:** Department of Microbiology, Prince of Wales Hospital, 30-32 Ngan Shing Street, Shatin, New Territories, Hong Kong

## Abstract

*Background*. The admission screening of methicillin-resistant *Staphylococcus aureus* (MRSA) by rapid molecular assay is considered to be an effective method in reducing the transmission of MRSA in intensive care unit (ICU). *Method*. The admission screening on patients from ICU once on their admissions by BD GeneOhm MRSA assay has been introduced to Prince of Wales Hospital, Hong Kong, since 2008. The assay was performed on weekdays and reported on the day of testing. Patients pending for results were under standard precautions until the negative screening results were notified, while contact precautions were implemented for MRSA-positive patients. In this study, we compared the MRSA transmission rate in molecular screening periods (2008 to 2010) with the historical culture periods (2006 to 2007) as control. *Results*. A total of 4679 samples were tested; the average carriage rate of MRSA on admission was 4.45%. By comparing with the historical culture periods, the mean incidence ICU-acquired MRSA infection was reduced from 3.67 to 1.73 per 1000 patient bed days. *Conclusion*. The implementation of admission screening of MRSA with molecular method in intensive care unit could reduce the MRSA transmission, especially in the area with high MRSA prevalence situation in Hong Kong.

## 1. Introduction

Methicillin-resistant strain *S. aureus* (MRSA) is a major cause of nosocomial infections; it causes infections with clinical manifestations ranging from pustules to sepsis and even death [1]. MRSA is frequently encountered in health-care settings and represents over 50% of isolates from hospital-acquired *S. aureus* in some North American hospitals [2]. Most transmissions occur through the contaminated hands of healthcare workers in hospital settings. Early screening of patients for MRSA nasal carriage is an effective infection control strategy to identify those patients that require isolation. However, the utility of active surveillance screening has been evaluated in many studies, and its effectiveness is still controversial [3, 4]. This controversy may be attributed to the slow turnaround time of the conventional culture method. Recently, many commercial available molecular assays have been developed; they provide a rapid tool for laboratory to shorten the turnaround time of the screening and reduce the time for resolution of MRSA carrier status within a day. Currently, the evidence in supporting MRSA universal screening on admission by molecular method is mixed and inconclusive. In fact, the effectiveness of screening depends on the prevalence of MRSA, the resources available for testing, and infection control policy.

In Hong Kong, MRSA is known to be endemic in hospitals. The incidence of MRSA clinical isolates was 0.5/100 deaths and discharges in 2000, and the carriage rate on entry to intensive care units was 12.1% [5, 6]. The incidence of hospital-transmitted MRSA infections was 0.26–0.29/1000 patient bed days from 2009 to 2011 in Prince of Wales Hospital, Hong Kong. With the high prevalence of MRSA and the rapidity at which MRSA infection can spread, the capability of providing screening results of MRSA carriage on the day of admission represents a definite advantage for infection control programs. A rapid screening could maximize the utilization of infection control resources. It assists in the earlier isolation of positive patients, allows early infection control strategies, and hence reduces the likelihood of transmission.

## 2. Methods

### 2.1. Hospital Setting

Prince of Wales hospital (PWH) is a 1,400-bed public hospital in Hong Kong affiliated to the Chinese University of Hong Kong. The adult intensive care unit (ICU) in Prince of Wales Hospital consists of 20 intensive care beds. It is made up for medical, surgical, neurological, and trauma patients.

### 2.2. Workflow and Study Period

Before the introduction of rapid molecular assay, MRSA screening in our ICU was performed by culture method. A new rapid molecular assay for admission screening has been implemented in the ICU since January 2008. Since then, all patients admitted to ICU were screened for MRSA by the molecular method once on admission. The subsequent weekly MRSA screening is still performed by culture method. The molecular screening test was performed by BD GeneOhm MRSA assay (Becton Dickinson), the test was available from Monday to Friday except public holidays, the samples cut-off time was 3:00 pm, and reports were printed to ICU before 6:00 pm. The samples that received outside normal working hours were kept at 4°C until processing.

The review period of the intervention in this study was from Jan 2008 to December 2010; the MRSA carriage rates and ICU-acquired MRSA infection rates were compared to the historical culture period from Jan 2006 to Dec 2007 as control.

### 2.3. Screening by Culture Method

Copan swabs taken from nasal or multiple sites were inserted into nutrient broth (Oxoid) supplemented with 7% NaCl and incubated in ambient air at 30°C overnight. After incubation, 10 *μ*L of the broth was subcultured on in-house prepared mannitol agar (Oxoid) with oxacillin and incubated at 37°C for 48 hours. Suspected MRSA colonies were confirmed by standard microbiology identification procedures.

### 2.4. Screening by Molecular Method

The molecular screening was performed by BD GeneOhm MRSA PCR assay. At the time of testing, the previous version of the assay using glass beads for bacterial lysis was used. Briefly, the BBL CutlureSwab for nasal swab was placed in a buffer tube and vortexed for 1 minute. The cell lysate was transferred to a lysis tube and then centrifuged at 14,000–21,000 g for 5 minutes. The supernatant was discarded using a sterile fine-tip transfer pipette without touching the pellet. After adding fresh sample buffer, the lysate was vortexed again for 5 minutes and spun down. The lysis tube was then heated to 95°C for 2 minutes and then put on a cooling block. The PCR was performed in SmartCycler II and analysed according to the manufacturer's procedures. The positive and negative results could be reported on the day of testing, if the samples were with inhibition for PCR; the indeterminate result was reported and another sample for culture is recommended.

### 2.5. Infection Control Policy for MRSA in ICU

Newly admitted patients in intensive care unit were under standard precautions until the MRSA screening results by molecular method were notified. Contact precautions were implemented for MRSA-positive patients including those who are placed in single room isolation with standard contact precautions, designated equipments, decolonization regimens, and antimicrobial soap for bathing.

### 2.6. Definition

ICU-acquired MRSA infection was defined as the patient developed any type of MRSA infections after 48 hours of ICU admission and had not been colonized or infected with MRSA before ICU admission.

MRSA infections were expressed as number of infections per 1000 patient bed days and analyzed according to different phases. Culture phase was defined as the period before the implementation of rapid molecular screening (Jan 2006 to Dec 2007), and PCR phase was defined as the period after the implementation of rapid molecular screening (Jan 2008 to Dec 2010).

## 3. Results

### 3.1. Prevalence of MRSA on Admission

In total, 3271 and 4679 samples were tested in culture and PCR phase, respectively. Forty-five samples in culture phase and 211 samples in PCR phase were positive (Table 1). The average MRSA carriage rate on admission in culture-phase was 1.38% and in PCR-phase was 4.45%.

### 3.2. ICU-Acquired MRSA Infection

**Figure 1:**
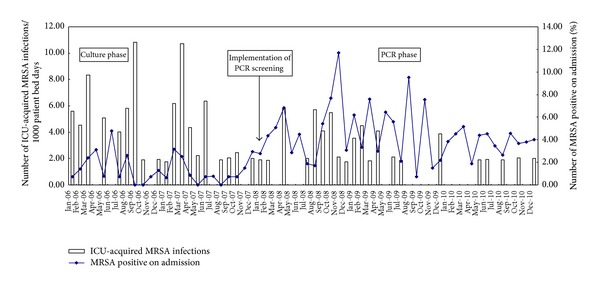
MRSA transmission in ICU during culture-phase and PCR-phase and the prevalence of MRSA on admission during the same periods.

In culture-phase, forty-three patients acquired MRSA infections in ICU, whereas only thirty-two patients acquired MRSA infections during PCR-phase. Overall, the mean incidence of MRSA transmission was 3.67 per 1000 patient bed days during the culture-phase and 1.73 per 1000 patient bed days during the PCR-phase. The reduction was 1.94 per 1000 patient bed days. The results were shown in Figure 1. Analyzing the data by months with the Mann-Whitney *U* test, the difference of MRSA transmission between culture and PCR phases was found to be statistically significant (*P* < 0.05).

## 4. Discussion

The control of spread of methicillin-resistant *Staphylococcus aureus* (MRSA) infection and colonization has become one of the most important issues in hospital settings. With the high mortality of MRSA infections and prolonged ICU stay with acquired MRSA infections, many interventions have been made to reduce MRSA transmission in hospitals. Reliable and rapid detection of MRSA-colonized patients is essential for the successful infection control measure to reduce transmission in hospitals. The implementation of rapid screening by molecular method is one of the effective methods to achieve this goal [7]. With the advance of technology, the promise of PCR can provide a short turnaround time report from sample to results reporting; thus, it allows earlier identification of MRSA carriers and may subsequently reduce MRSA transmission, especially in critical care units. Due to the recent availability of commercial real-time PCR assay for MRSA screening, we applied and were granted funding from hospital management to implement a rapid molecular admission screening for all newly admitted ICU patients since 2008. A nasal swab taken from patients admitted to ICU was screened for MRSA by BD GeneOhm MRSA assay. The test has been commenced for 5 years and is still ongoing. In this study, we reviewed three-year data from 2008 to 2010 and compared them to the historical culture period from 2006 to 2007 as control. The results showed that the mean incidence of acquired MRSA infections in ICU for patients who were screened by molecular method compared with patients who were screened by culture method was reduced from 3.67 to 1.73 per 1000 patients bed days and the finding was statistically significant (*P* < 0.05), while at the same period the carriage rate increased from 1.38% to 4.45%. Many studies have been published on the effectiveness of rapid screening and the results were contradictory. Hardy et al. showed a significant reduction in MRSA transmission between PCR and culture method, but Jeyaratnam et al. did not find a significant difference in the MRSA transmission and acquisition rates between PCR and culture methods [8, 9]. A recent review by Polisena et al. found small differences in the MRSA transmission rates between screening using PCR and culture methods [10]. The contradictory findings can be explained by the fact that rapid molecular screening is only one of the contributing components of MRSA infection control program, and it is difficult to accurately determine its relative contribution to the overall outcome. The success of the screening program relies on the efficacy of the infection control measures including hand hygiene compliance, environmental cleansing and disinfection, contact isolation and cohorting of patients, dedicated use of medical equipments decolonization regimens, judicious use of antibiotics, and staff education. Harbarth et al. found that rapid screening had no impact on a purely surgical ICU; however, Cunningham et al. showed that there is a reduction in MRSA transmission in a mixed units of medical, surgical, and neurosurgical ICU [11, 12]. Thus, the effectiveness of rapid screening is more effective in the multidiscipline ICU. Moreover, rapid screening is more effective to reduce the MRSA transmission in the area with high prevalence. In low MRSA prevalence countries, for example, The Netherlands and Scandinavian countries, policy of preemptive isolation of patients with high risk of MRSA carriage appears to be critical, but it is not applicable in high prevalence area, like Hong Kong; preemptive patient isolation is considered to be cumbersome for hospital staff and may ultimately reduce the quality of patient's care. We believed that the rapid admission screening with standard precautions may be the useful choice in our ICU setting. One of the major concerns was resources and expense of molecular method compared to conventional culture. However, their usefulness is still under investigation. In this study, we demonstrated that the MRSA-acquired infection in ICU is significantly decreased; hence, the overall resources for patient care's are definitely reduced. Another concern raised by frontline staff is the potential increase in number of patients placed under precautions. This is not always a problem as the screening results could always be completed within 24 hours in weekdays. Overall, the falling of MRSA burden should allow a subsequent reduction in financial expenditure and the amount of staff time spent dealing with MRSA infections. The former should offset the increased cost of the test.

The turnaround time of the GeneOhm MRSA assay is fast, it can be completed within two hours, and the test is easy to perform; the overall performance of the assay is satisfactory. The number of indeterminate cases by the assay due to the presence of inhibitors was 12.4% which is similar to Rajan's study but higher than other studies [13, 14]. The reason for the high unresolved rate may be due to the crude glass beads lysis method of the assay. In the new version of the assay launched in 2011, the cell lysis has been changed to enzymatic lysis by achromopeptidase; the unresolved rate was reduced to around 1%, and the overall performance was improved a lot [15].

A new MRSA strain from human and livestock carrying a *mec*A gene variant, *mec*C or *mec*A_LGA251_, was identified in Europe [16, 17]. The strain can be isolated by routine culture method and is phenotypically resistant to cefoxitin which is *mec*A-negative. This animal-associated MRSA strain has been shown to be pathogenic for humans. Therefore, the epidemiologic situation should be carefully monitored to prevent the spread of this strain in human population and, in particular, into health care settings. However, such monitoring is made difficult because the commercial available PCR detection assays for screening cannot detect the strain with *mec*C; thus, they can be escaped from the current molecular screening detection. Commercial companies should be aware of this and revise their kits to improve their performance.

The limitation of this study was that only small numbers of samples were compared with PCR and culture methods in the early evaluation periods and no confirmation of PCR by culture method was done after the service live run. Samples with false positive and negative will be reported in this setting. In our laboratory, we could only provide the rapid screening in the weekdays if the service can be available 7 days a week. The overall turnaround time can be further decreased; hence the outcome may be more pronounced, but the overall expenditure is definitely increased. Except for the colonization pressure, other potential confounding factors, such as antibiotics usage, changes in MRSA epidemiology, and seasonal variation, were not adjusted in the analysis.

In conclusion, we demonstrated that the implementation of rapid admission screening of MRSA by molecular method with standard precautions policy is an effective approach in reducing the MRSA transmission in intensive care unit, especially in the area with high MRSA prevalence.

## Figures and Tables

**Table 1:** Yearly results of MRSA screening on admission and number of positive cases of ICU-acquired MRSA infections during culture-phase and PCR-phase.

Year	MRSA PCR results on admission			ICU-acquired MRSA infections		
	No. of positive	No. of negative	Total no. of samples	No. of positive cases	MRSA transmission/1000 patient bed days	
Culture phase						
2006	25	1593	1618	24		4.00
2007	20	1633	1653	19		3.34

Total	45	3226	3271	43	Average	3.67

PCR phase						
2008	76	1474	1550	16		2.57
2009	77	1555	1632	11		1.81
2010	58	1439	1497	5		0.81

Total	211	4468	4679	32	Average	1.73

## References

[1] Centers for Disease Control and Prevention (2001). Methicillin-resistant *Staphylococcus aureus* skin or soft tissue infection in a state prison. Mississippi, 2000. *Morbidity and Mortality Weekly Report*.

[2] National Nosocomial Infections Surveillance System (2002). National Nosocomial Infections Surveillance (NNIS) System Report, data summary from January 1992 to June 2002, issued August 2002. *American Journal of Infection Control*.

[3] Harbarth S, Hawkey PM, Tenover F, Stefani S, Pantosti A, Struelens MJ (2011). Update on screening and clinical diagnosis of meticillin-resistant *Staphylococcus aureus* (MRSA). *International Journal of Antimicrobial Agents*.

[4] McGinigle KL, Gourlay ML, Buchanan IB (2008). The use of active surveillance cultures in adult intensive care units to reduce methicillin-resistant *Staphylococcus aureus*-related morbidity, mortality, and costs: a systematic review. *Clinical Infectious Diseases*.

[5] You JHS, Ip DNC, Wong CTN, Ling T, Lee N, Ip M (2008). Meticillin-resistant *Staphylococcus aureus* bacteraemia in Hong Kong. *Journal of Hospital Infection*.

[6] Ho P-L (2003). Carriage of methicillin-resistant *Staphylococcus aureus*, ceftazidime-resistant gram-negative bacilli, and vancomycin-resistant enterococci before and after intensive care unit admission. *Critical Care Medicine*.

[7] Creamer E, Dolan A, Sherlock O (2010). The effect of rapid screening for methicillin-resistant *Staphylococcus aureus* (MRSA) on the identification and earlier isolation of MRSA-positive patients. *Infection Control and Hospital Epidemiology*.

[8] Hardy K, Price C, Szczepura A (2010). Reduction in the rate of methicillin-resistant *Staphylococcus aureus* acquisition in surgical wards by rapid screening for colonization: a prospective, cross-over study. *Clinical Microbiology and Infection*.

[9] Jeyaratnam D, Whitty CJM, Phillips K (2008). Impact of rapid screening tests on acquisition of meticillin resistant *Staphylococcus aureus*: cluster randomised crossover trial. *British Medical Journal*.

[10] Polisena J, Chen S, Cimon K, McGill S, Forward K, Gardam M (2011). Clinical effectiveness of rapid tests for methicillin resistant *Staphylococcus aureus* (MRSA) in hospitalized patients: a systematic review. *BMC Infectious Diseases*.

[11] Harbarth S, Fankhauser C, Schrenzel J (2008). Universal screening for methicillin-resistant *Staphylococcus aureus* at hospital admission and nosocomial infection in surgical patients. *The Journal of the American Medical Association*.

[12] Cunningham R, Jenks P, Northwood J, Wallis M, Ferguson S, Hunt S (2007). Effect on MRSA transmission of rapid PCR testing of patients admitted to critical care. *Journal of Hospital Infection*.

[13] Rajan L, Smyth E, Humphreys H (2007). Screening for MRSA in ICU patients. How does PCR compare with culture?. *Journal of Infection*.

[14] Dalla Valle C, Pasca MR, de Vitis D, Marzani FC, Emmi V, Marone P (2009). Control of MRSA infection and colonisation in an intensive care unit by GeneOhm MRSA assay and culture methods. *BMC Infectious Diseases*.

[15] Patel PA, Ledeboer NA, Ginocchio CC (2011). Performance of the BD GeneOhm MRSA achromopeptidase assay for real-time PCR detection of methicillin-resistant *Staphylococcus aureus* in nasal specimens. *Journal of Clinical Microbiology*.

[16] Laurent F, Chardon H, Haenni M (2012). MRSA harboring *mec*A variant gene *mec*C, France. *Emerging Infectious Diseases*.

[17] Petersen A, Stegger M, Heltberg O (2013). Epidemiology of methicillin-resistant *Staphylococcus aureus* carrying the novel *mec*C gene in Denmark corroborates a zoonotic reservoir with transmission to humans. *Clinical Microbiology and Infection*.

